# Southern Surgical and Gynecological Association

**Published:** 1893-12

**Authors:** 


### SOUTHERN SURGICAL AND GYNECOLOGICAL ASSOCIATION.

The meeting of this body which was held in New Orleans on the 14th, 15th and 16th inst., sustained fully the former position in the interest of papers read and the enjoyment offered the members by the courtesies extended to them by the local profession and by the citizens.

There was an important feature added by those in charge of the Charity Hospital in opportunities given for surgical operation by several surgeons who were in attendance. Dr. Hunter McGuire, of of Richmond, did a suprapubic cystotomy, Dr. W. T. Briggs, of Nashville, trephined a subject for epilepsy and performed lithotomy on the cadaver by his bilateral operation. Dr. H. A. Kelly, of the Johns Hopkins Institute, executed a hysterectomy for uterine fibroid of large size, and Dr. Joseph Price, of Philadelphia, made an abdominal section for removal of diseased tubes and ovaries.

The two latter named illustrated their respective modes of closing the abdominal incision, the former uniting the peritoneum, fascia and skin by separate lines of suture, the latter bringing all the tissues together by a single interrupted suture.

The paper which may prove as the most important in its results was that of Dr. B. E. Hadra, of Galveston, Texas, upon epilepsy. He detailed cases in which the electric current had served to localize that portion of the brain involved and the subsequent operations confirmed the diagnosis.

A paper was read by Dr. L. McLane Tiffany, of Baltimore, upon "Intracraneal Neurectomy and Removal of Gasserian Ganglion" which opens up a new field of practical relief in this class of cases.

The presentation by Dr. W. B. Rogers, of Memphis, of his results with the button of Dr. J. B. Murphy, of Chicago, for attaching the gall-bladder to the intestinal canal and in end to end union of the intestine, afforded additional evidence of this procedure for anastomosis.

The only papers read by Georgians were that upon "Treatment of Gun-shot Wounds," by Dr. Willis F. Westmoreland, and that upon "Operative Procedure for Carcinomatous Tumors of the Breast," by Dr. J. McFadden Gaston.

A paper was submitted to the council upon "Management of Cases after Celiotomy," by Dr. W. A. Crow, of Atlanta, with his application for membership, which received favorable consideration; and will appear in the published transactions.

There were twenty-eight new members elected at this meeting, Dr. Crow being one of this number. These will contribute materially to the working force of this body in the future. It is generally conceded in this country and abroad that the scientific work of the previous meetings of this Association compared favorably with that of any other body, and it is confidently believed that the papers contributed at the recent meeting fully sustain the high standard of merit already attained by the Southern Surgical and Gynecological Association.

The next place of meeting will be Charleston, S. C., and the officers elected are as follows: President, Dr. Cornelius Kollock, of Cheraw, S. C.; First-Vice President, Dr. A. B. Miles, of New Orleans; Second Vice-President, Dr. J. B. S. Holmes, of Atlanta; Secretary, Dr. W. E. B. Davis, of Birmingham. J. McF. G.

